# Composites Based on Hydroxyapatite and Whey Protein Isolate for Applications in Bone Regeneration

**DOI:** 10.3390/ma14092317

**Published:** 2021-04-29

**Authors:** Dagmara Słota, Magdalena Głąb, Bożena Tyliszczak, Timothy E. L. Douglas, Karolina Rudnicka, Krzysztof Miernik, Mateusz M. Urbaniak, Paulina Rusek-Wala, Agnieszka Sobczak-Kupiec

**Affiliations:** 1 Faculty of Materials Engineering and Physics, Department of Materials Science, Cracow University of Technology, 31-864 Krakow, Poland; magdalena.glab@doktorant.pk.edu.pl (M.G.); bozena.tyliszczak@pk.edu.pl (B.T.); kmiernik@pk.edu.pl (K.M.); agnieszka.sobczak-kupiec@pk.edu.pl (A.S.-K.); 2 Materials Science Institute (MSI), Lancaster University, Lancaster, UK; t.douglas@lancaster.ac.uk; 3 Engineering Department, Lancaster University, Lancaster LA1 4YW, UK; 4 Department of Immunology and Infectious Biology, Faculty of Biology and Environmental Protection, University of Lodz, 90-237 Lodz, Poland; karolina.rudnicka@biol.uni.lodz.pl (K.R.); mateusz.urbaniak2@unilodz.eu (M.M.U.); paulina.rusek@unilodz.eu (P.R.-W.)

**Keywords:** hydroxyapatite, ceramic biomaterials, whey protein isolate, composites

## Abstract

Hydroxyapatite (HAp) is a bioactive ceramic with great potential for the regeneration of the skeletal system. However, its mechanical properties, especially its brittleness, limit its application. Therefore, in order to increase its ability to transmit stresses, it can be combined with a polymer phase, which increases its strength without eliminating the important aspect of bioactivity. The presented work focuses on obtaining organic–inorganic hydrogel materials based on whey protein isolate (WPI) reinforced with nano-HAp powder. The proportion of the ceramic phase was in the range of 0–15%. Firstly, a physicochemical analysis of the materials was performed using XRD, FT-IR and SEM. The hydrogel composites were subjected to swelling capacity measurements, potentiometric and conductivity analysis, and in vitro tests in four liquids: distilled water, Ringer’s fluid, artificial saliva, and simulated body fluid (SBF). The incubation results demonstrated the successful formation of new layers of apatite as a result of the interaction with the fluids. Additionally, the influence of the materials on the metabolic activity according to ISO 10993-5:2009 was evaluated by identifying direct contact cytotoxicity towards L-929 mouse fibroblasts, which served as a reference. Moreover, the stimulation of monocytes by hydrogels via the induction of nuclear factor (NF)-κB was investigated. The WPI/HAp composite hydrogels presented in this study therefore show great potential for use as novel bone substitutes.

## 1. Introduction

Advances in civilization’s changes in lifestyle and aging societies in the developed world are resulting in an increasing occurrence of the so-called diseases of the 21st century. These non-communicable diseases (NCDs) are chronic, lifestyle-related and non-infectious to others. There are four main NCDs, namely cardiovascular diseases (such as heart attack and stroke), cancer, chronic respiratory diseases (such as chronic obstructive pulmonary disease and asthma) and diabetes. However, hypertension, Alzheimer’s disease and osteoporosis are also increasingly common. According to the WHO 2014 report, non-communicable diseases are responsible for almost 70% of premature deaths [1,2]. For the studies presented, osteoporosis is particularly significant, as it is an enormous and growing public health problem and consequently the most common skeletal ailment. It is estimated that currently more than 200 million people are affected, and that it causes over 8.9 million fragility fractures each year [3,4]. It affects every third woman and every fifth man aged over 50. The higher prevalence in women is related to the change in hormone metabolism after the menopause, and due to lower levels of estrogen [5]. It is a progressive disease, manifested by a decrease in bone density, which weakens it as well as increasing the risk of fracture. After a bone break, chronic pain and reduced ability to perform basic activities may occur [6]. The treatment of osteoporosis uses known antiresorptive drugs and new anabolic options which are being constantly proposed. However, concerns regarding side effects as well as long-term effects remain under-investigated, and contribute to the substantial under-treatment of patients [7]. Therefore, new, innovative methods of treatment and prevention are being sought, preferably those containing active components and exhibiting bioactivity towards damaged tissues.

A widely used material with impressive bioactive properties and with a high chemical and crystallographic similarity to the inorganic phase of bone is hydroxyapatite (HAp). It is applied as an orthopedic biomaterial and in dentistry to replace hard tissues. Particularly interesting is its biocompatibility, ability to bind to natural bone, its porous structure and its ability to enable the growth of the surrounding tissues [8,9]. The feature that distinguishes HAp from other materials used in implantology is osteoinduction, a process that induces osteogenesis, leading to the formation of new bone [10,11]. This is an extremely important aspect in the treatment of osteoporosis. In the osteoporotic state, there is an excessive activity of osteoclasts, the bone-degrading cells, resulting in skeletal destruction and a dangerous loss of bone mass [12,13]. HAp improves the response of osteoblasts, thus maintaining the balance between osteoblasts and osteoclasts [14,15]. However, HAp, despite its advantages, has several disadvantages that limit its usability. These are brittleness, low tensile strength and fracture toughness. Therefore, such an implant will not be able to withstand high stress and will be destroyed quickly [16]. The solution to these problems, while maintaining the bioactive character of the material, is to create a composite with an elastic phase, which exhibits a higher flexibility than HAp [17].

One of the types of polymer scaffolds with several potential advantages in bone regeneration are hydrogels [18]. These consist of hydrophilic polymer chains arranged in a three-dimensional (3D) space. The hydrophilic nature of the scaffolds allows it to provide a nutritional environment suitable for the growth of endogenous cells. Due to their structural similarity to the natural extracellular matrix (ECM), they show a promising ability to encapsulate drugs, cells or bioactive particles. Thus, they function as carriers of active substances which can facilitate local controlled release [19,20,21]. Hydrogels are characterized by high absorbency, tunable viscoelasticity, and permeability to oxygen and essential nutrients. Furthermore, and most importantly, they show excellent integration with surrounding tissues, which reduces the chances of inflammatory reactions [22]. However, traditional hydrogels are bulk gels consisting of inert polymers that are not bioactive and have low mechanical strength [23]. Therefore, a combination of a hydrogel with a hydroxyapatite phase would make it possible to obtain a composite with enhanced strength through the ability of the polymer to carry the stresses, and with superior bioactive properties thanks to the presence of the HAp phase.

Whey is a by-product of the cheese production process, obtained from bovine milk [24]. The whey protein isolated from it contains peptides, glycopeptides and other protein glycoconjugates with immunomodulating properties such as α-lactalbumins, β-lactoglobulins and components promoting bone mineralization such as calcium, lactates, and phosphates [25,26]. Commercially processed whey protein occurs in three main forms, as a concentrate (WPC), an isolate (WPI) and a hydrolysate (WPH) [27]. The following studies were based on WPI. WPI is a processed product, purified to remove fat and lactose, containing over 90% protein. Purification can be carried out by microfiltration followed by ultrafiltration and spray drying or ion exchange, after which the product concentration and spray drying takes place [28]. WPI demonstrates good water solubility and undergoes the process of gelation after heating. This results in the formation of protein bonds inside the structure of the hydrogel, which eliminates the need for the use of additional chemical crosslinking agents [29]. Due to its impressive biocompatibility and sensitivity to pH, WPI hydrogel can potentially be used as a carrier of biomolecules, or as a drug delivery system for the controlled release of medicines [30]. Therefore, WPI appears to be a suitable material from which to create a modified hydrogel with potential applications in tissue regeneration.

This work is devoted to the fabrication and in vitro characterization of composite hydrogels based on WPI protein and hydroxyapatite. In order to precisely analyze the obtained biomaterials, they were subjected to incubation tests in simulated body fluid (SBF), artificial saliva and Ringer’s fluid. Furthermore, cell biological analysis was performed by analyzing the cytotoxicity against mouse fibroblast cell line L-929 and the monocyte activation towards THP1-Blue™ NF-κB cells derived from the THP-1 human monocytic leukemia cell line. To the best of our knowledge, there are no studies on the preparation, physicochemical properties and bioactivity of the materials obtained in the manner presented in this study.

## 2. Materials and Methods

### 2.1. Materials

WPI (BiPRO) containing 97.7% protein and 75% β-lactoglobulins (according to the manufacturer’s specification) was purchased from Davisco Foods International Inc (Eden Prairie, MN, USA). The calcium acetate monohydrate (Ca(CH_3_CO_2_)_2_·H_2_O), sodium phosphate dibasic (Na_2_HPO_4_), ammonium phosphate monobasic (NH_4_H_2_PO_4_), calcium nitrate tetrahydrate (Ca(NO_3_)_2_·4H_2_O) and ammonia water (NH_4_OH, 25%) used in the HAp synthesis were obtained from Sigma-Aldrich (Darmstadt, Germany). For biological assay L-929 (CCL-1™), mouse fibroblasts (ATCC, Manassas, VA, USA), THP1-Blue™ NF-κB cells (InvivoGen, San Diego, CA, USA), fetal bovine serum (FBS) (HyClone Cytiva, Marlborough, MA, USA), penicillin, strepto-mycin, 3-(4,5-dimethylthiazol-2-yl)-2,5-diphenyltetrazolium bromide (MTT), dimethyl sulfoxide, monocytes stimulated with β-glucan from *Saccharomycces cerevisiae* (*S. cerevisiae*) (Sigma-Aldrich, Darmstadt, Germany), trypsin-EDTA solution (Gibco, Waltham, MA, USA), 4-(2-hydroxyethyl)-1-piperazineethanesulfonic acid (HEPES), blasticidin and QUANTI-Blue™ (InvivoGen) were used.

### 2.2. Preparation of Hydroxyapatite

HAp, representing the ceramic phase of the composite hydrogels, was obtained via two wet precipitation methods. For this purpose, in the first method (method A), performed at room temperature, 80 mL NH_4_H_2_PO_4_ (0.36 mol/L) was added to 520 mL distilled water, and then a few drops of ammonia water were added in order to obtain a pH value > 10. Then, 200 mL Ca(NO_3_)_2_ solution at a concentration of 0.6 mol/L was added dropwise under constant stirring at a rate of 1 drop/s. The second method (method B) was performed at boiling point. A few drops of ammonia water were added to 80 mL Na_2_HPO_4_ solution at a concentration of 0.32 mol/L in order to obtain an alkaline pH value (pH > 10) of the mixture. Then, 520 mL distilled water was added and the mixture was heated to boiling point. When the boiling point was reached, 200 mL (CH_3_COO)_2_Ca (0.128 mol/L) was added dropwise under constant stirring at a rate of 1 drop/s. After each reaction was completed, the obtained HAp powder was aged for 24 h at room temperature, and then the sludge was washed thoroughly with distilled water to achieve a neutral pH and finally dried at 104 °C for 4 h in a laboratory dryer.

### 2.3. Preparation of the Composite Hydrogels

The WPI-based hydrogel was obtained as a result of a gelation reaction. In total, 60 mL distilled water was added to 40 g WPI by stirring intensively on a magnetic stirrer. In this way, a WPI solution of 40% (*w*/*w*) concentration was obtained. The reaction was carried out at room temperature. After the complete dissolution of the WPI, the solution was left for approximately 2 h in order to allow the resulting foam to settle.

In order to obtain stable composites, the ceramic phase A, at three different concentrations (5%, 10%, 15% weight percentages) was added to Eppendorf tubes containing the WPI solution. All of the samples were quickly mixed using a vortexer for approximately 2 min in order to distribute the phases evenly throughout the volume. Then, to prevent the sedimentation and stabilization of the composites, the Eppendorf tubes were placed in a water bath at 90 °C for 7 min. In this way, cylindrical composites with a diameter of 1 cm and a height of 3 cm were obtained. The operations were repeated for ceramic phase B. WPI hydrogels without HAp were also synthesized as control samples. All of the samples were sterilized using a UV lamp. The compositions of each composite are presented in Table 1.

### 2.4. X-ray Diffraction Analysis

The phase composition and crystallinity of the two kinds of HAp powders were determined using the X-ray diffraction (XRD) method. For this purpose, a Rigaku SmartLab X-ray Difractometer (Wilmington, MA, USA) was used. The measurement was performed at a voltage of 40 kV and 30 mA in a 2θ range of 10–60° and at a step size of 0.002° 2θ.

### 2.5. Fourier Transform Infrared Spectroscopy Analysis

Fourier transform infrared spectroscopy (FT-IR) was used to analyze the hydroxyapatite and the composition of hydrogels before and after incubation, and to determine the functional groups present in the materials. The Thermo Scientific Nicolet iS5 FTIR spectrophotometer equipped with iD7 ATR (Loughborough, UK) was used for this purpose. The device was equipped with a high-performance, reflective optical system and was supplied with a monolithic diamond crystal ATR. This crystal enabled a high optical contact between the sample and the diamond, which lead to a good resolution. The spectra were recorded in the wavelength range 4000–400 cm^−1^, at room temperature.

### 2.6. Incubation In Vitro

The obtained dried hydrogel samples with an initial mass of 1 g were incubated for 14 days in artificial biological fluids and distilled water at a constant temperature of 37 °C. Three solutions—namely artificial saliva, SBF and Ringer’s solution—were prepared; their compositions are shown in Table 2, Table 3, Table 4. The ingredients were mixed according to a specified order, and each component was added after the complete dissolution of the previous ones. The SBF solution was prepared at a constant temperature of 36 ± 1.5 °C.

### 2.7. Swelling Capacity

The swelling capacity of the composites was studied in order to determine the amount of fluid that they could absorb at a given time. The process was carried out in distilled water and selected artificial biological fluids. The samples were discs, which were cut out of the prepared composite hydrogel (see Section 2.3). The discs had an initial mass of 1 g. The swelling ratio (*S_w_*) was calculated using the following Equation (1):

(1) $$S_{w}=\frac{\left(W_{t}-W_{0}\right)}{W_{0}}\cdot 100\%$$

where *W_t_* is the weight of the swollen hydrogel sample and *W*_0_ is the initial sample weight [31].

### 2.8. Morphology Analysis

The analysis of the surface morphology by scanning electron microscopy was performed in order to determine the topography of the hydroxyapatite and hydrogel composites before and after the incubation period. The study enabled the detection of potential deposits formed on the surface of the samples as a result of interaction with individual ions of artificial biological fluids.

A Jeol 5510LV Scanning Electron Microscope with a EDS IXRF System detector (Freising, Germany) was used for this purpose. Before the measurement, the samples were thoroughly dried at 36.5 °C for 48 h and sputtered with a nano-layer of gold.

### 2.9. Stability Measurements

The stability of the emulsions was evaluated using MultiScan MS20 DataPhysics Instruments (Charlotte, NC, USA). This technique consisted in detecting the transmission of monochromatic NIR radiation at 100 nm, using a transmission detector that measures the radiation transmitted by the sample. The measurement enabled the determination of both the descent rate of the particles in the suspension and their average diameter. The sedimentation rate of hydroxyapatite particles in water and WPI solution was determined. The study was based on the observation of the behavior of the HAp suspension over time. The rate of sedimentation and the obtained transmission provides information on the homogeneity of the substance. The rate of sedimentation varies depending on the monodispersing or polydispersing nature of the suspension. The measurements were carried out at 25 °C.

### 2.10. In Vitro Cytocompatibility

#### 2.10.1. Cell Culture

The influence of composite hydrogels on cell metabolic activity was assessed according to ISO 10993-5:2009 (Biological evaluation of medical devices—Part 5: Tests for in vitro cytotoxicity) toward reference L-929 (CCL-1™) mouse fibroblasts obtained from the American Type Culture Collection. Prior to the experiments, fibroblasts were cultured in Roswell Park Memorial Institute (RPMI)-1640 medium supplemented with 10% heat-inactivated fetal bovine serum (FBS) and standard antibiotics: penicillin (100 U/mL) and streptomycin (100 µg/mL). The cells were incubated at 37 °C in a 5% CO_2_ atmosphere in a humidified cell incubator until the formation of the cell monolayer. For the subculturing, confluent cell monolayers were detached from the bottom of the culture flasks with 0.25% trypsin-EDTA solution and resuspended in RPMI-1640 medium. Following the centrifugation (400× *g*, 10 min), the cell pellet was resuspended in fresh medium, and the cell viability and density were assessed using a counting Bürker chamber (Blaubrand, Wertheim, Germany) and a trypan blue exclusion assay (Avantor Performance Materials, Gliwice, Poland).

#### 2.10.2. Direct Contact Cytotoxicity Assay

The L-929 fibroblasts adjusted to a density of 2 × 10^5^ cells/mL were transferred into 96-well culture plates (20,000 cells/per well) (Nunclon Delta Surface, Nunc, Rochester, NY, USA) and incubated overnight in standard conditions at 37 °C. After incubation, the L-929 cell cultures were observed using an inverted contrast phase microscope (Motic AE2000, Xiamen, China) in order to confirm that the confluent monolayer had formed. Suspensions of the investigated pieces of composite hydrogels (WPI0—the control sample, WPI A5, WPI A10, WPI A15, WPI B5, WPI B10, WPI B15) corresponding to one-tenth of the well surface area were prepared under sterile conditions. The cell culture medium was replaced with 100 µL fresh RPMI-1960 medium, and pieces of the composite hydrogels were added to selected wells in six replicates. The cell cultures in the medium without the tested samples were used as a positive control for cell viability. After overnight incubation, the condition of the cell monolayers was verified under an inverted contrast phase microscope. In order to quantify the cell viability, 20 µL 3-(4,5-dimethylthiazol-2-yl)-2,5-diphenyltetrazolium bromide (MTT) was added to each well and incubation was carried out for another 4 h. In the next step, the plates were centrifuged at 450× *g* for 10 min, the remaining liquid was removed, and the formazan crystals were dissolved with 100 µL dimethyl sulfoxide. The absorbance was determined spectrophotometrically using the Multiskan EX plate reader (Thermo Scientific, Waltham, MA, USA) at 570 nm.

### 2.11. Monocyte Activation

THP1-Blue™ NF-κB cells derived from the human THP-1 monocyte cell line were used to examine whether the WPI present in the hydroxyapatite and hydrogels stimulate monocytes by the induction of nuclear factor NF-κB. The primary cell cultures were passaged two times in RPMI 1640 medium supplemented with heat-inactivated 10% FBS, 25 mM 4-(2-hydroxyethyl)-1-piperazineethanesulfonic acid (HEPES), penicillin/streptomycin solution (100 U/mL; 100 µg/mL), 2 mM glutamine and 100 µg/mL normocin. Then, THP1-Blue™ NF-κB monocytes were re-cultured in selective RPMI 1640 medium additionally containing 10 µg/mL blasticidin in a density below 1 × 10^6^ cells/mL at 37 °C, and in a humidified atmosphere containing 5% CO_2_. The experiments were conducted as described previously [32,33]. Briefly, monocytes were adjusted to the optimal density (1 × 10^6^ cells/mL) and distributed equally in each well of the 96-well plate (200 µL/well). Then, as described in Section 2.10.2, hydroxyapatites and sections of the hydrogels were added to selected wells, in four replicates. The following positive and negative controls of NF-κB activation were included: monocytes stimulated with β-glucan from *Saccharomycces cerevisiae* (*S. cerevisiae*) and monocytes incubated in complete medium, respectively. In order to quantify the monocyte activation, 20 µL of each supernatant was transferred to and mixed with 180 µL QUANTI-Blue™ substrate reagent to detect the activity of secreted embryonic alkaline phosphatase (SEAP). The absorbance was measured after 4 h incubation at 650 nm on the Multiskan EX plate reader (Thermo Scientific, Waltham, MA, USA).

## 3. Results

### 3.1. X-ray Diffraction Analysis of HAp

**Figure 1:**
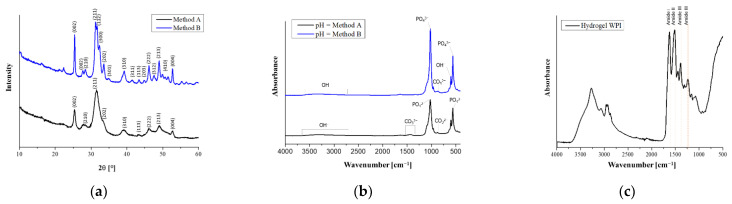
(**a**) XRD diffractograms of hydroxyapatite obtained by methods A and B; (**b**) FTIR spectra of hydroxyapatite obtained by methods A and B; (**c**) FT-IR spectra of WPI hydrogel.

The result of the XRD analysis of the powders consisted of two diffractograms, which are shown in Figure 1a. Although the obtained diffractograms vary considerably, especially in the intensity of the individual bands, the analysis showed that both ceramic materials were pure, and the only phase identified by X-rays in the examined powders was HAp. The graph for powder A obtained by X-ray analysis suggests that the substance obtained was more amorphous than for powder B, which appeared to be more crystalline, because of the more intense background peaks. According to the International Center for Diffraction Data (No. 9-432), XRD reflections were assigned to the hexagonal structure of HAp [34].

### 3.2. Fourier-Transform Infrared Spectroscopy Analysis

#### 3.2.1. FT-IR Analysis of HAp

The spectroscopic spectra of the hydroxyapatite powders obtained are shown in Figure 1b. There are many chemical groups in the FTIR spectrum of HAp that are characteristic for this material. Infrared absorption spectrum analysis showed the presence of phosphate groups in both investigated HAp powders, which is evidenced by clear bands in the range 550–1050 cm^−1^. For sample A, the double band in the wavelength range 555 cm^−1^ to 565 cm^−1^ is associated with a triply degenerated bending mode of the O−P−O in the PO_4_^3−^ groups, which occupied two sites in the crystal lattice; in material B, the values of 600 cm^−1^ and 565 cm^−1^ are corresponding. The presence of sharp and splitting peaks at this range indicated a high degree of crystallinity, which is particularly noticeable for HAp B [35]. Characteristic for HAp, the intense peaks associated with asymmetrical P−O tensile vibrations are attributed to the values 965 cm^−1^ and 1026 cm^−1^ for material B and 1011 cm^−1^ for sample A [36]. The weak peaks of 1620 cm^−1^, shown in both graphs, are more intense in B, corresponding to the presence of OH^−^ associated with the HAp molecules [35]. The wide peak for values 2900–3640 cm^−1^ indicate the occurrence of valence vibrations of the OH^−^ group; these are also present in B, 2900–3540 cm^−1^, although they possess less intensity. Those broad absorption bands are attributed to the presence of H_2_O associated with the HAp molecules [37]. However, in sample A, there are peaks that do not coincide with diagram B. These differences are probably caused by the use of other substrates and a different way of performing the synthesis. These are values at 1320 cm^−1^ and 1480 cm^−1^, the presence of which is caused by atmospheric synthesis. They suggest the presence of a bending mode of carbon ions [38]. The respective peaks with their assigned function groups are summarized in Table 5.

#### 3.2.2. FT-IR Analysis of the WPI Matrix and WPI/HAp Composites

**Figure 2:**
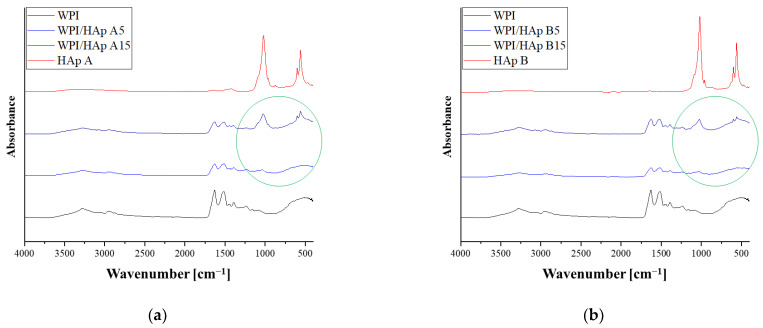
FT-IR spectra of composites with different Hap contents indicated by a green circle: (**a**) WPI/HAp A; (**b**) WPI/HAp B.

The FT-IR analysis was performed both for the composites with different contents of the ceramic phase and for WPI hydrogel without the addition of HAp. The results of the analysis are presented in the Figure 2. In the case of unmodified WPI hydrogel (Figure 1c), a wide absorption band was observed in the wavenumber range 3600–3000 cm^−1^ (with a maximum at about 3278 cm^−1^), which was assigned to stretching vibrations of the −OH and −NH groups [39]. Then, in the range 3000–2850 cm^−1^, a band of relatively low intensity corresponding to C−H stretching vibrations of carbonyl groups of triglycerides (lipids present in dairy products) was identified. The next bands are associated with peptide bonds, and are characteristic of whey proteins. The maximum at 1632 cm^−1^ is a characteristic band of the primary amide group of proteins (−CO−NH_2_). The band at 1520 cm^−1^ corresponds to the secondary amide group of proteins (−CO−NH) [40]. The range 1400–1200 cm^−1^ was attributed to the amide III [41]. The observed characteristic band at around 1040 cm^−1^ was assigned to the stretching of C−O vibrations [42]. These vibrations are most probably derived from saccharides present in whey protein, which suggests that, despite purification, traces of lactose were still present in the WPI. The characteristic vibration range for the specific groups is presented in Table 6.

The FT-IR spectra of composites containing different amounts of the ceramic phase are presented in Figure 2a for hydroxyapatite obtained by method A, and in Figure 2b for hydroxyapatite obtained by method B. The analysis was conducted for composites containing 5 and 15% ceramic phases. The obtained results were compared with the spectrum of pure hydroxyapatite and unmodified WPI hydrogel. In the graphs, a green circle indicates the presence of groups characteristic of hydroxyapatite in the composite material. In the case of composites containing a 5% ceramic phase, a new absorption band in the wavelength range 1010–1025 cm^−1^ was observed on the spectrum. This band has a relatively low intensity, and the range corresponds to asymmetrical P−O tensile vibrations. For a composite containing 15% hydroxyapatite, a new band was also observed in this range with a much higher peak intensity. Moreover, in the case of a composite containing 15% HAp, a band at a wavelength of about 560–590 cm^−1^—characteristic of the triply degenerate bending of PO_4_^3−^ (O−P−O)—was also observed. Analogous results were obtained both for HAp obtained using method A and HAp obtained using method B. Therefore, it was found that the intensity of the absorption bands originating from the P−O stretching vibrations and from the triply degenerate bending of PO_4_^3−^ increases with the percentage of the ceramic phase in the composite.

### 3.3. Incubation In Vitro

#### 3.3.1. Electroanalytical Analysis—Conductivity

**Figure 3:**
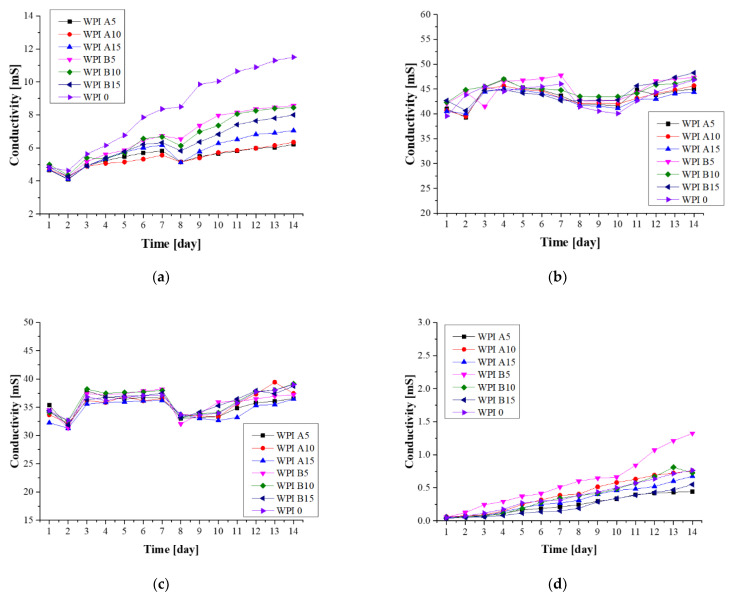
Conductivity analysis of hydrogels during a 14-day incubation in fluids simulating the biological environment: (**a**) incubation in artificial saliva; (**b**) incubation in SBF; (**c**) incubation in Ringer’s fluid; (**d**) incubation in distilled water.

Using conductivity measurements, the electrolyte conductivity was measured (Figure 3). The conductivity changes with the concentration of ions during incubation in fluids simulating the living environment. These changes indicate the interaction between biomaterial and incubation fluids, and the possibility of ion exchange. Changes were observed in all of the samples, the largest of which were in artificial saliva, in which the samples were decomposed during incubation. In each case, the curves in the graph increased. The fact that the total decomposition of the sample was observed during incubation in artificial saliva is directly related to the greatest changes in the conductivity values. This indicates that samples degrade under the given conditions. The increase in conductivity in this case is the result of an increase in the ion concentration due to decomposition. This is particularly noticeable for the WPI 0 sample, which is the polymer matrix itself. The situation is different for incubation in SBF, where changes were also observed; however, they are relatively small. This proves the relative stability of materials in this liquid. The changes observed in Ringer’s fluid are probably dependent on the fact that the partial resorption of the material was observed; however, due to the presence of certain amounts of calcium and phosphate in the composition of the fluid, remineralization on the surface of the biomaterial was also observed. The greatest stability of samples was observed for incubation in distilled water. Such stability is associated with the absence of other ions in the solution with which the biomaterial could interact.

#### 3.3.2. Electrochemical Analysis—Potentiometry

**Figure 4:**
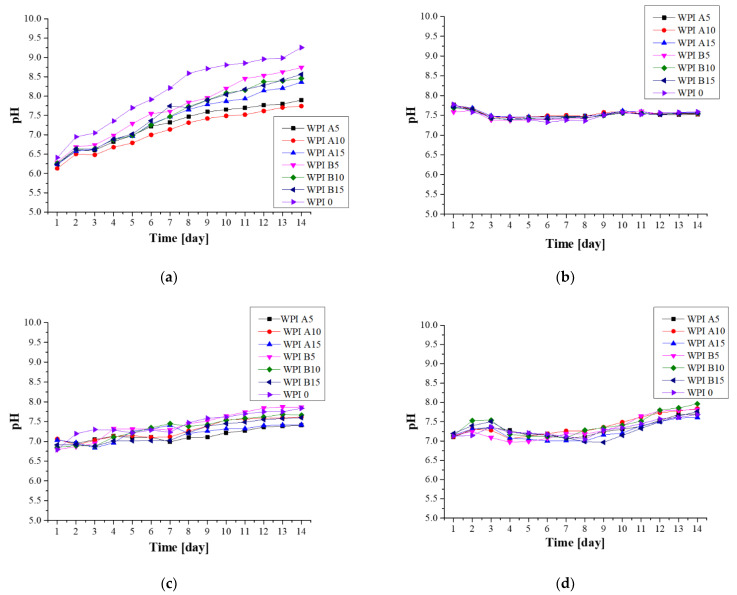
Potentiometry analysis of the hydrogels during a 14-day incubation in fluids simulating the biological environment: (**a**) incubation in artificial saliva; (**b**) incubation in SBF; (**c**) incubation in Ringer’s fluid; (**d**) incubation in distilled water.

A potentiometric analysis was performed in order to determine the pH change of the solutions in which the samples were incubated (Figure 4). This measurement allows the determination of the stability of the material in the chosen solution, and is closely related to conductivity analysis. Repeatedly, the greatest changes were observed during incubation in artificial saliva, in which the samples degraded. A significant change in pH value was observed in relation to the initial value of 6.2. As a result of the total degradation, the ions and compounds released from the composites caused a significant increase in pH. The SBF incubation pH values remained stable between 7.3 and 7.8 throughout the incubation period. With respect to the remaining incubation fluids, the smallest changes were again observed in SBF, which indicates the stability of the sample in this fluid. Additionally, the formation of new apatite layers on the surface, especially in SBF but also in Ringer’s solution, may limit the degradation process and thus also limit the rapid change of the pH value.

#### 3.3.3. Swelling Capacity

**Figure 5:**
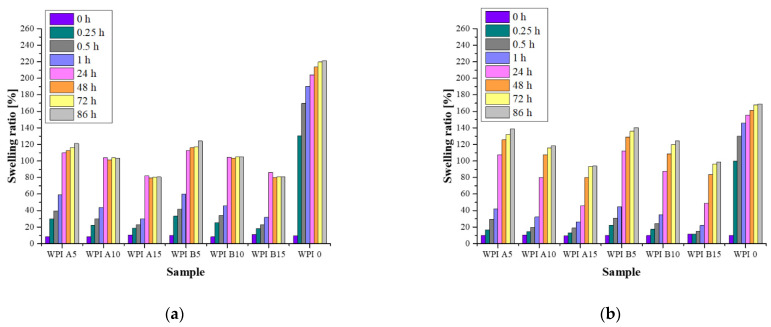

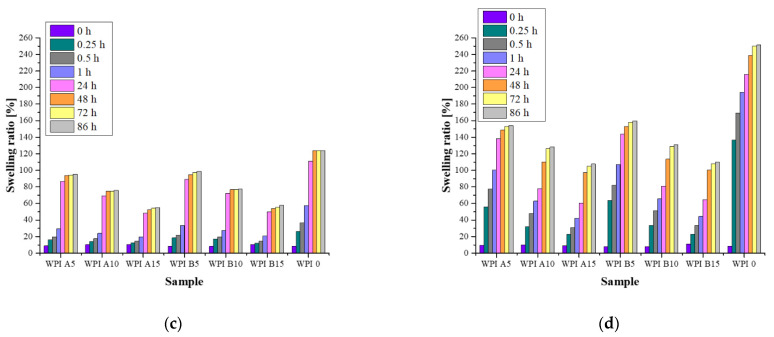
Swelling ability of hydrogels during a 14-day incubation in fluids simulating the biological environment: (**a**) incubation in artificial saliva; (**b**) incubation in SBF; (**c**) incubation in Ringer’s fluid; (**d**) incubation in distilled water.

The swelling capacity was determined for unmodified WPI hydrogel and for those with added hydroxyapatites A and B (Figure 5). As a result, the swelling effect of the hydrogel materials was confirmed for all of the tested samples. The analysis was performed in four fluids: artificial saliva, SBF, Ringer’s fluid and distilled water. It was observed that, in all of the fluids, unmodified WPI hydrogels had the highest sorption capacity. On the other hand, swelling coefficients decreased with increasing proportion of the ceramic phase, e.g., for WPI A5 and WPI A15 these are 109% and 82% (24 h, artificial saliva), respectively. This is most probably related to the presence of the ceramic phase, which fills the free spaces between the polymer chains while limiting the possibility of fluid sorption. In the case of WPI hydrogel without the addition of HAp, there are free spaces between the chains into which the fluid easily penetrates. An analogous relationship was found for all fluids. Moreover, small differences in the swelling coefficients were also observed in each fluid for hydrogels with the addition of HAp obtained by different methods. A higher sorption capacity was found in samples containing hydroxyapatite obtained by method A. According to the previously presented results of the XRD analysis, this powder was characterized by a lower degree of crystallinity than HAp B. Therefore, it can be assumed that the higher sorption capacity of the composites is related to the physicochemical properties of the ceramic phase present in the polymer matrix. Most probably, HAp A—which is more amorphous—was more easily washed out of the polymer, and the remaining free spaces were filled by the absorbed liquid. It is also important that the swelling rates of hydrogels immersed in distilled water are the highest among the fluids studied. This is related to the fact that, in the remaining fluids, there are different ions that can form additional transverse bonds between the polymer chains. As a result, the cross-linking density of the polymer matrix increases while the free space available for the fluids is reduced, which translates into a lower sorption capacity. An increase in the swelling coefficients was observed for all of the samples over time. However, after about 48 h, the next changes were relatively small. On the other hand, the highest swelling rate was observed in the first 24 h. The exception was the unmodified WPI hydrogel, for which the swelling coefficients reached significantly higher values after just 15 min.

### 3.4. Morphology Analysis

#### 3.4.1. Hydroxyapatite Morphology

**Figure 6:**
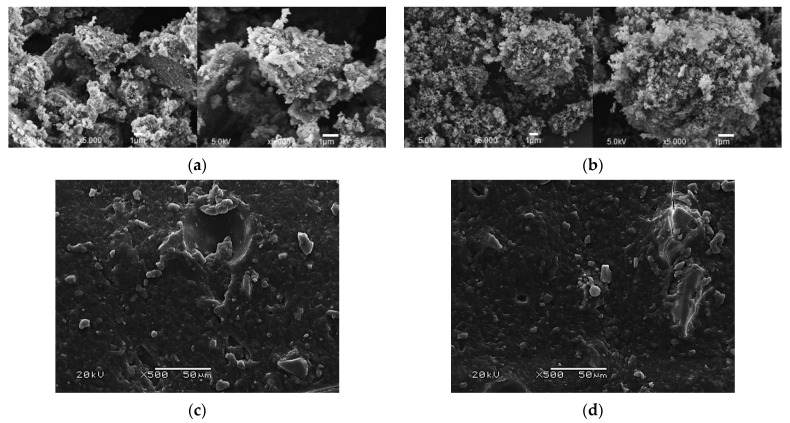

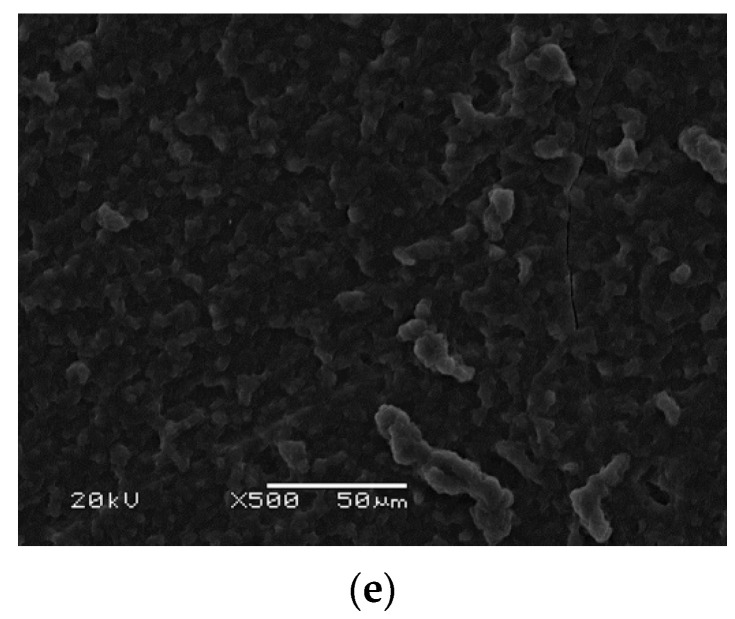
Morphology analysis of the HAp powders and composite materials before the incubation period: (**a**) SEM images of hydroxyapatite obtained by method A; (**b**) SEM images of hydroxyapatite obtained by method B; (**c**) SEM image of the WPI/HAp A5 composite; (**d**); SEM image of the WPI/HAp B5 composite; (**e**) SEM image of a pure WPI hydrogel matrix.

In order to determine the morphologies of the obtained calcium phosphates, an SEM analysis was performed. The SEM images of the HAp obtained by method A are presented in Figure 6a, and those for method B are shown in Figure 6b. On the basis of the analysis, it was observed that the particles of hydroxyapatite obtained in method A are characterized by a morphology resembling that of powder. HAp B, on the other hand, is characterized by a spherical, flocculated morphology. It is also noticeable that HAp B is a powder consisting of larger agglomerates than HAp A. This information regarding grain size significantly influenced the subsequent incubation stages of the materials, as the durability and resorption of the hydrogel in an in vitro environment depend upon it.

#### 3.4.2. Hydrogels’ Morphology before Incubation

Figure 6c,d shows the surfaces of the composite hydrogels before the incubation in biological fluids. The surface of the hydrogel WPI itself is also presented (Figure 6e). For the composite hydrogels, the presence of HAp powders in the materials is clearly visible, while the surface of the cross-linked WPI itself is relatively smooth. Perceptibly, with an increase in the proportion of the ceramic phase in the hydrogel, the amount of crystals observed during the measurement increases.

#### 3.4.3. Hydrogels’ Morphology after Incubation

**Figure 7:**
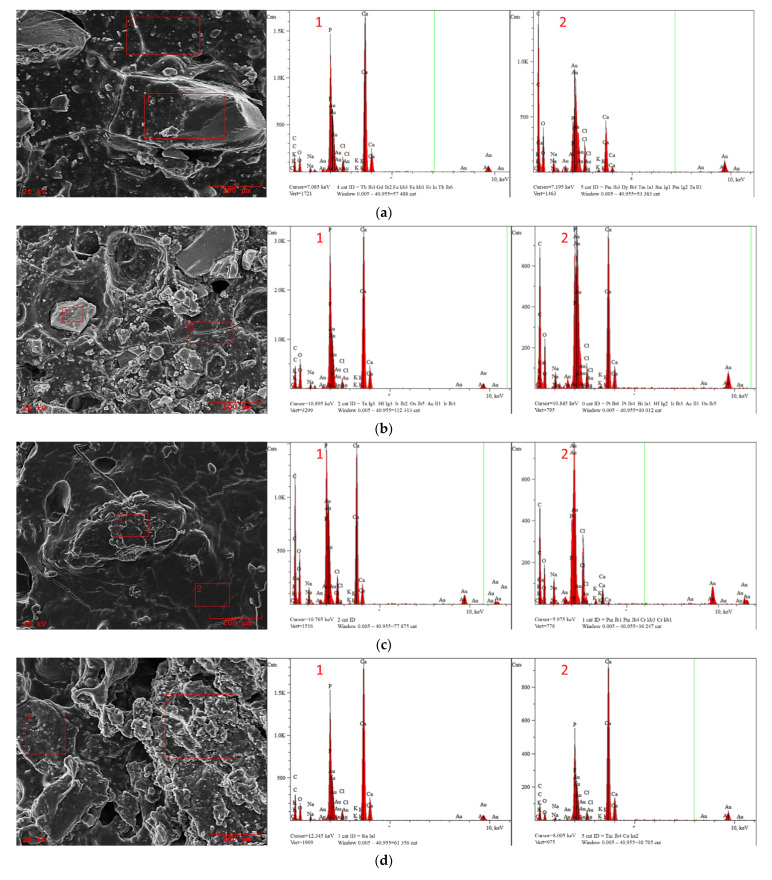

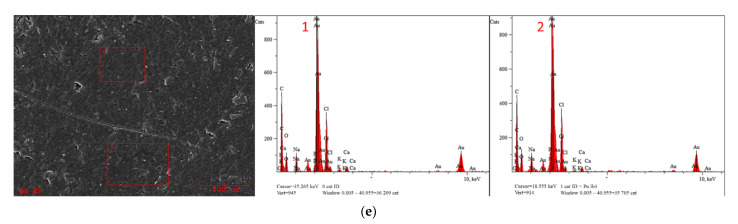
SEM morphology and EDS microanalysis of the composites and the pure WPI matrix after incubation in SBF: (**a**) WPI A5; (**b**) WPI A15; (**c**) WPI B5; (**d**) WPI D15; (**e**) pure WPI hydrogel.

The hydrogels were incubated for a period of 2 weeks in the selected artificial biological fluids. Figure 7a,b presents a comparison of the surface morphology of the WPI A5 and WPI A15 samples together with an EDS microanalysis after incubation in SBF. An analogous comparison was made for WPI B5 and WPI B15 (Figure 7c,d). Similar results were obtained for both cases. In SBF, in the hydrogels with a lower content of the ceramic phase, after incubation the spots where practically pure polymer matrix was present were still visible, and the EDS microanalysis showed only trace amounts of the elements P and Ca. However, in the case of samples with a higher proportion of the ceramic phase, a pure polymer matrix itself could not be seen. The entire surface of the biomaterial was covered with new crystals. In addition, the EDS microanalysis of these samples showed the highest intensity of the peaks for P and Ca in the areas studied.

The appearance of new apatite layers and crystals on the surface of the incubated samples is the most desirable result. Such biomineralisation is a testament to the bioactivity of the WPI/HAp hydrogel due to its confirmation of the interaction between the material and the incubating fluid.

An analogous measurement was carried out for the WPI matrix itself, without ceramic reinforcement. The obtained result is presented in Figure 7e. The lack of apatite crystals on the surface confirms that the WPI protein itself does not promote the formation of apatite layers. Consequently, one would not expect it to demonstrate bioactivity when implanted in bone.

Therefore, the ability to form mineralized apatite layers on the surface of incubated hydrogels is fully dependent on the presence of HAp in the biomaterial. Accordingly, in materials containing a greater proportion of the ceramic phase, the ability to induce mineralization by new apatite formations will be significantly higher, which would be expected to improve the biocompatibility.

Moreover, a detailed EDS analysis concerning the presence of specific elements is presented in Table 7. The presence of Au in each of the samples is connected with the necessity of gold sputtering before SEM analysis. The presence of C and O atoms was noticeable in each sample, which is related to the composition of the polymer matrix. The detection of Mg, Na and Cl ions after the incubation indicates the desired reactions between artificial physiological fluid and biomaterial. The analysis of the elemental composition of hydrogels, especially the amounts of Ca and P, shows that slightly larger amounts of these elements after the incubation period are observed in samples containing HAp B. In the WPI 0 sample, the amount of these desired elements was negligible.

Analogously to the previous samples, the morphology and elemental composition of hydrogels incubated in Ringer’s solution and distilled water were determined. In the case of samples incubated in artificial saliva, the full degradation of the material made SEM/EDS analysis impossible. This process was probably caused by the acidic nature of the fluid. Furthermore, the total decomposition may be associated with the hydrolytic degradation of the polymer, as the ions present in artificial saliva may contribute to the acceleration of this process [43,44].

**Figure 8:**
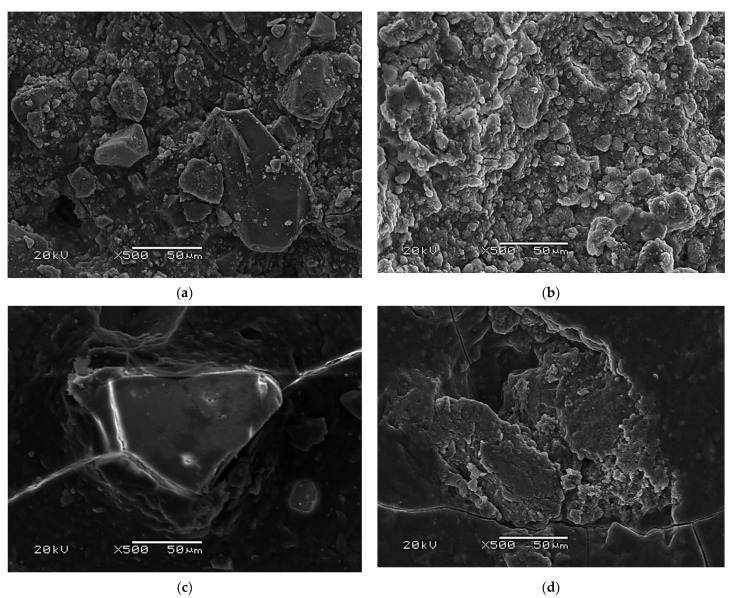
(**a**) SEM morphology of WPI A15 after incubation in Ringer’s fluid; (**b**) SEM morphology of WPI B15 after incubation in Ringer’s fluid; (**c**) SEM morphology of WPI A15 after incubation in distilled water; (**d**) SEM morphology of WPI B15 after incubation in distilled water.

However, the partial degradation of the polymer matrix was also observed for the samples incubated in Ringer’s solution. The SEM images of the selected hydrogels after incubation in Ringer’s solution and distilled water are presented in Figure 8.

On the surface of the samples incubated in Ringer’s fluid, ceramic crystals can be observed; however, this is probably the hydroxyapatite placed in the hydrogel before the incubation process began. This conclusion is based on the fact that during the incubation period, the biomaterial significantly reduced its volume. This is most likely due to the partial degradation of the protein matrix due to the slightly acidic nature of the fluid. In the case of incubation in distilled water, there are only single points indicating the presence of the ceramic phase. It can therefore be concluded that the formation of new apatite layers depends on both the percentage of the ceramic phase and the incubation environment. Distilled water, in which no Ca and P ions are present, is an environment which interacts poorly with the tested biomaterial. As the content of the selected ions increases, these interactions also increase, as was observed in the case of Ringer’s fluid and the SBF solution.

### 3.5. Stability Measurements

The stability of the hydroxyapatite suspension was determined by measuring the sedimentation rate of HAp particles in distilled water and in a 40% WPI solution. The measurement was performed for both HAp A and HAp B under the conditions presented in Table 8.

**Figure 9:**
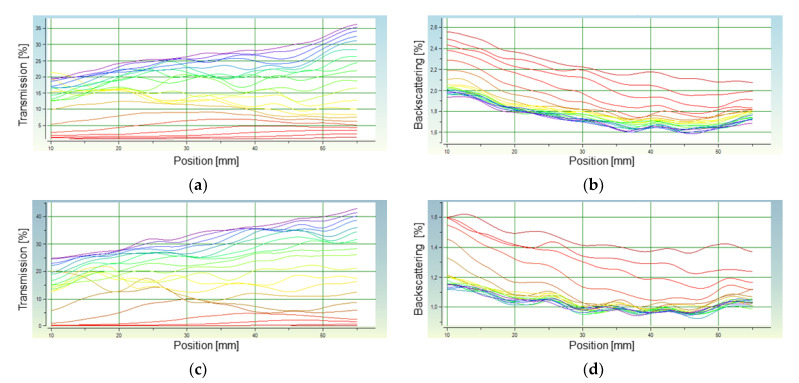
Analysis of the stability of the HAp A and HAp B suspensions in distilled water: (**a**) transmission of HAp A; (**b**) backscattering of HAp A; (**c**) transmission of HAp B; (**d**) backscattering of HAp B.

The results of the analysis performed in distilled water are presented in Figure 9a,b (HAp A) and Figure 9c,d (HAp B). In both cases, 20 measurements were taken with an interval of 15 s within 5 min. The transmission value increasing with time indicates a sedimentation process taking place. This increase occurs to the greatest extent at the highest position value; it indicates the gradual clarification of the suspension in the upper part of the measuring vessel. A significant increase in the backscattering at low position values, i.e., at the lower part of the measuring vessel, indicates the accumulation of falling HAp particles. In the case of HAp B, the transmission values are higher than for HAp A. Because the larger the particle diameter, the higher the sedimentation rate, it can be assumed that HAp B is characterized by a larger size of individual particles, which fall faster. In the case of HAp A, the changes in the transmission are also irregular, but the curves in the graph are less wavy than for HAp B. The course of the curves in the graph proves that sedimentation does not occur uniformly in the entire volume. Therefore, it can be concluded that the analyzed suspension is more monodispersive for HAp A than for B.

An analogous measurement was also performed for both HAp preparations in a 40% WPI solution. In this case, 20 measurements were taken with an interval of 90 s. Based on the analysis carried out, a migration front was determined for all of the samples, which made it possible to determine the sedimentation rate of the HAp particles in distilled water and a WPI solution. The sedimentation rates determined on the basis of the migration front graphs are presented in Table 9.

The sedimentation rate is directly proportional to the size and mass of the falling particles, and inversely proportional to the liquid viscosity. This means that th esedimentation increases with particle size and weight, and with the decreasing viscosity of the liquid in which they are dispersed. The sedimentation rate of HAp in 40% WPI solution is much lower than the rate of their sedimentation in distilled water, which is connected with the higher viscosity of the WPI solution. In the case of composites consisting of a polymer and ceramic phase, this is an important fact because increasing the viscosity of the WPI solution offers the possibility of obtaining a more homogeneous system than in the case of a low viscosity solution, in which rapid particle sedimentation occurs. The sedimentation rate of HAp particles in the WPI solution is 0.05016 mm/min for HAp A and 0.1101 mm/min for HAp B, respectively. This means that the gelation process that takes place in 7 min is fast enough to prevent the significant sedimentation of HAp particles. In the case of sedimentation in distilled water, the HAp particles fall much faster, and the sedimentation rate values indicate a faster drop of HAp B particles. Using the relationship between particle size and falling speed, the analysis also allowed the determination of the average diameter of the hydroxyapatite particles in the suspension. The diameters were determined for the HAp samples in distilled water (assumed parameters: particle density 3.2 g/cm^3^; solvent density 0.9982 g/cm^3^; solvent viscosity 1.002 mPa; particle volume Conc. HAP A: 1.279%, HAp B: 1.328%). The average diameter of the HAp A particles was ⌀ = 0.7245 µm, while HAp B was ⌀ = 0.9631 µm. These results are consistent with the conclusions drawn from the SEM images, according to which, in the case of single grains, the HAp A particles are smaller in size.

### 3.6. The Viability of Cells in the Milieu of the Biocomposites

**Figure 10:**
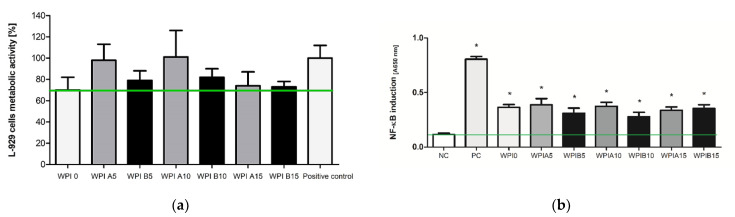
(**a**) The viability of murine fibroblasts L-929 after 24 h incubation with WPI modified HAp A and HAp B biocomposites, evaluated using the 3-(4,5-dimethylthiazol-2-yl)-2,5-diphenyltetrazolium bromide (MTT) reduction assay according to ISO-10993-5:2009. The cells incubated without composites served as a positive control of the viability (100%). The data are presented as mean ± SD for the three separate experiments. The green line indicates the minimum level (70%) of the cells’ metabolic activity required to recognize the biomaterial as non-cytotoxic at the in vitro level; (**b**) NF-κB induction in THP1-Blue™ monocytes incubated for 24 h with WPI modified HAp A, and HAp B biocomposites. The cells incubated without composites served as a negative control of the monocyte’s activation (NC), and monocytes stimulated with *S. cerevisiae* β-glucan served as the positive control (PC). The data are presented as the mean ± SD for the four separate experiments. The green line indicates the physiological level (0.114 ± 0.013) of the non-stimulated monocytes. * *p* values (<0.05) calculate in comparison to the untreated cell cultures.

The influence of biocomposites containing WPI hydrogels with HAp A or HAp B on the viability of the reference L-929 mouse fibroblasts was assessed in the MTT reduction assay, which verified the metabolic activity of mitochondrial dehydrogenases in the environment of the tested substances. In this test, the biochemical activity of mitochondrial enzymes positively correlated with cell viability. As shown in Figure 10a, the viabilities of murine fibroblasts exposed to WPI 0, WPI 5A, WPI 10A and WPI 15A were found to be 70 ± 12%, 98 ± 15%, 101 ± 25% and 74 ± 13%, respectively. A statistical analysis showed that WPI modified with HAp A and HAp B did not reduce significantly the metabolic activity of L-929 cells when compared with the physiological viability of the control cells.

A decrease in the metabolic activity was observed only after the incubation of L-929 cells with WPI0 and WPI 15A; however, the cell viability was not reduced below 30% compared to the control cells. WPI modification with hydroxyapatite B—WPI 0, WPI 5B, WPI 10B and WPI 15B—resulted in the viability of L-929 cells equal to 70 ± 12%, 79 ± 9%, 82 ± 8%, 73 ± 5%, respectively. Moreover, it was shown that the mice fibroblasts exposed to WPI composites modified with HAp obtained using precipitation method B exhibited lower metabolic activity in comparison to the untreated cells or cells incubated in the milieu of HAp A-modified WPI composites.

### 3.7. The Biocomposite-Mediated Monocyte Activation

Because monocyte activity is crucial for the induction of a first (inflammatory) stage of the healing process, we attempted to evaluate the WPI-mediated influence of the biocomposites on the NF-κB induction in THP1-Blue™ monocytes. It was shown that all of the tested biocomposies containing WPI stimulated the activity of monocytes in comparison to the untreated cell cultures (*p* < 0.05 *), and this effect occurred regardless of modification with HAp A or HAp B (Figure 10b). In addition, when the results were analyzed with ANOVA with Tukey’s post-hoc test, it was shown that the WPI A5 (0.388 ± 0.056), WPI A10 (0.374 ± 0.036), WPI A15 (0.337 ± 0.030) and WPI B5 (0.310 ± 0.047), WPI B10 (0.279 ± 0.039) and WPI B15 (0.354 ± 0.034) biocomposites induced higher monocyte activation than that observed for the positive control (0.852 ± 0.015; *p* < 0.05 #).

## 4. Discussion

Composites based on polymers reinforced with HAp are materials known to be applied in the regeneration of the skeletal system. However, it seems crucial to choose an appropriate polymer phase, and a type of ceramic with suitable properties. In this study, two methods of ceramic synthesis were selected in order to produce two different hydrogel composite materials. Both presented methods resulted in the synthesis of ceramics which were confirmed to be HAp by FT-IR analysis. However, it was confirmed that their physicochemical parameters were slightly different. Depending on preferences, a material with a larger or smaller grain diameter can be obtained. The degree of crystallinity determined by the XRD also varied. The method used to obtain HAp B, due the elevated temperature at which it is conducted, results in a product with a higher degree of crystallinity than HAp A, which is obtained at room temperature. The difference in the density or grain size of the powders obtained was observed by SEM analysis and by measurements with the Multiscan MS20. The analysis of migration front and sedimentation rate clearly indicates a larger grain diameter for HAp B. Moreover, the powders were shown to be medium monodispersive. The sedimentation rates were also determined in WPI solutions in order to select the appropriate polymer concentration for hydrogel synthesis.

Both hydroxyapatite A and B were used to synthesize WPI hydrogels in order to assess the influence of equal types of these ceramics on hydrogel behavior. Despite slight differences in the physicochemical parameters of HAp, the hydrogels behaved similarly during incubation. A process of biomineralization was used to assesses the mineralization of hydrogels as a predictor of bioactivity, and to study the influence of the substrate composition on the process of biomineralization in in vitro conditions. Hydrogel materials containing the ceramic phase HAp A, the ceramic phase HAp B and no ceramic phase were examined. Four incubation fluids were selected, including distilled water, SBF, artificial saliva and Ringer’s fluid. It was determined that the WPI matrix itself does not demonstrate the desired bioactivity; however, the addition of the ceramic phase raises it. The strongest promotion of mineralization was observed for hydrogels WPI A15% and WPI B15% incubated in SBF. This is clearly visible in the pictures of the SEM analysis. The 15% composites showed the highest bioactivity, as the new apatite layers were more frequently observed there compared to the other samples. Furthermore, the EDS microanalysis indicated that after barely two weeks of incubation, there are no places without layers of apatite on the surface of the material. This effect is closely related to the nature and the composition of SBF, as the formation of new apatite layers in this liquid is highly dependent on the presence of calcium ions and their interactions [45]. Furthermore, the conductivity and potentiometry measurements indicated interactions between biomaterials and the incubation environment. The clear changes and increases in value in artificial saliva are associated with an acid reaction of this fluid, which was too aggressive for the protein matrix and caused its complete degradation. Minor changes in the case of SBF suggest the stability of hydrogels in this environment. Moreover, the swelling capacity analysis confirmed the potential use of composites in drug delivery systems. The highest swelling coefficients were observed for distilled water. In addition, a higher swelling capacity was shown for materials without a ceramic phase, which is related to the fact that there are more free spaces between the polymer chains. However, even a smaller increase observed for materials containing 15% ceramics is a promising outcome.

The conducted microbiological analysis also yielded satisfactory results and confirmed the safety and application potential of the presented materials, as biomaterials are considered safe on an in vitro level in the absence of biomaterial-mediated cytotoxic activity. In this study, we have shown that hydrogel materials consisting of WPI with a ceramic phase of HAp A or HAp B met the ISO 10993-5: 2009 criterion for maintaining the in vitro viability of at least 70% of the cells exposed to the biomaterial. Moreover, the presented results are consistent with the current results reported by other authors. The non-toxic nature of whey protein-based hydrogels was confirmed on the mouse pre-adipose 3T3-L1 and myoblast C2C12 cell lines [46]. Dziadek et al. showed that WPI-hydrogel modified with alpha-tricalcium phosphate (α-TCP), one of the most chemically reactive forms of bioactive calcium phosphate (CaP) ceramics, do not reduce the cell viability of human osteoblast-like MG-63 after 3 days of stimulation in a direct contact cytotoxicity assay. On the other hand, after a 7-day culture on WPI hydrogel modified with α-TCP, osteoblast-like cells showed higher metabolic activity [47]. The experiments of Gupta et al. showed that argonite-modified WPI hydrogels with different pore diameters promoted osteoblast proliferation in a resazurin reduction assay over a 3-week culture period [41]. Although the properties of HAp may appear similar because they share the same chemical formula, the way they are synthesized and processed may affect their biological properties. In our study, we also showed that WPI composites modified with HAp obtained by precipitation method B induced lower metabolic activity in fibroblast cultures, in comparison to untreated cells or cells incubated in the milieu of HAp A-modified WPI composites. It was shown previously by other authors that size, structure and pore diameters influence hydroxyapatite biocompatibility, and it was assumed that the differences in the HAp synthesis methods (A vs. B) influenced the viability of the target cells. Zhao et al. demonstrated that nanosized needle-shaped and plate-shaped hydroxyapatite induced higher cytotoxicity than sphere-shaped and rod-shaped hydroxyapatite [48].

Apart from the lack of cytotoxicity, newly developed composites should exhibit features favoring the healing process. Monocytes are the first immune cells that appear at the site of an injury. They are involved in healing processes, tissue rearrangement, and—due to secretion of cytokines and chemokines—they regulate immune-tissue homeostasis [49]. Injured tissue is rich in signals and cells that propagate the inflammatory response; this stage, although dynamic and acute, is crucial to attract and activate monocytes in the site of implantation. Moreover, monocytes attracted to local bone injuries transform into macrophages, which stimulates osteogenic cells to promote mineralization and induce osteogenesis [50,51]. The role for monocytes in the post-implantation processes and their activity is crucial for the clearance of damaged cells and dis-integrated tissues from the site of the injury. This process is facilitated when monocytes are already in an activated state, which might be obtained by biological modifications of the biocomposites. Recently, the ability of pathogen recognition receptor ligands to skew the immune response in a specific direction was shown. Khokhani and colleagues successfully used nucleic acid-based ligands as osteo-immunomodulators, which favor early osteogenic differentiation without inducing an exaggerated immune-cell–mediated response [52].

The immunomodulatory properties of whey proteins are known; however, we have shown, for the first time, that WPI hydrogels modified with HAp activate THP1-Blue^TM^ monocytes via the NF-κB transcription factor. WPI hydrogels modified with HAp A and HAp B may promote the regeneration process by monocyte migration and activation at the site of bone fraction/implantation. In order to fully address the question of whether WPI-mediated immunostimulation facilitates regeneration processes, further experiments will be conducted. The materials merit further study.

## 5. Conclusions

In this study, we fabricated composite hydrogels by a gelation method at an elevated temperature. Two types of hydroxyapatites, slightly different from each other in terms of their physicochemical properties, were used for this purpose. The combination of bioactive ceramics and WPI with a very high biological value provided a material with satisfactory properties. The results obtained from the physicochemical and biological characterizations presented in this study suggest that such hydrogels constitute a potentially good material for bone tissue regeneration. Thus, this work presents the idea of using low-cost materials with high potential. Depending on the place of implantation and the desired resorption, both of them can be applied.

Further work should focus on further biological studies concerning the influence on bone cell activity and on the sorption potential of the presented hydrogels, and thus their use as a drug and active substances carrier in targeted therapy.

## Figures and Tables

**Table 1:** Percentage share of ceramics in hydrogels WPI/HAp.

Sample Symbol	Ceramic Content (%)
WPI 0	-
WPI A5	5
WPI A10	10
WPI A15	15
WPI B5	5
WPI B10	10
WPI B15	15

**Table 2:** Composition of the Ringer’s solution.

Component	Amount (g/L)
NaCl	8.600
KCl	0.300
CaCl_2_·H_2_O	0.480

**Table 3:** Composition of the artificial saliva.

Component	Amount (g/L)
NaCl	0.400
KCl	0.400
CaCl_2_·H_2_O	0.795
Na_2_HPO_4_·H_2_O	0.780
Na_2_S·9H_2_O	0.005
CH_4_N_2_O	1.000

**Table 4:** Composition of the SBF.

Component	Amount (g/L)
NaCl	8.035
NaHCO_3_	0.355
KCl	0.225
K_2_HPO_4_·3 H_2_O	0.231
MgCl_2_·6 H_2_O	0.311
1M HCl	39 mL
CaCl_2_	0.292
Na_2_SO_4_	0.072
Tris	6.118

**Table 5:** IR bad assignment of HAp.

Type of Hydroxyapatite	Wavenumber (cm^−1^)	Peak Assignment
HAp A	2900–3640	Band corresponding to H_2_O absorption
	1620	Band corresponding to H_2_O absorption
	1480	Carbonate ions
	1320	Carbonate ions
	1011	Asymmetric Stretching mode of P-O
	840	Carbonate ions
	565	Triply degenerate Bending of PO_4_^3−^ (O−P−O)
	555	Triply degenerate bending of PO_4_^3−^ (O−P−O)
HAp B	2900–3540	Band corresponding to H_2_O absorption
	1620	Band corresponding to H_2_O absorption
	1026	Asymmetric Stretching mode of P−O
	965	Asymmetric Stretching mode of P−O
	845	Carbonate ions
	600	Triply degenerate bending of PO_4_^3−^ (O−P−O)
	565	Triply degenerate bending of PO_4_^3−^ (O−P−O)

**Table 6:** IR band assignment of WPI.

Wavenumber (cm^−1^)	Peak Assignment
3600–3000	Stretching vibrations of −OH and −NH
3000–2850	Stretching vibrations of C−H
1632	Amide I
1520	Amide II
1400–1200	Amide III
1040	Stretching vibrations of C-O

**Table 7:** Elemental composition of hydrogels after incubation in SBF.

Sample	Spot	Atomic Percentage (wt.%)
WPI A5	1	C: 12.8, O: 17.1, Na: 1.1, Mg: 0.5, P: 16.9, Cl: 2.5, K: 0.2, Ca: 38.9, Au: 10.0
	2	C: 46.7, O: 22.4, Na: 1.6, Mg: 0.4, P: 5.6, Cl: 2.6, K: 0.4, Ca: 10.8, Au: 9.5
WPI A15	1	C: 19.1, O: 24.4, Na: 1.1, Mg: 0.5, P: 16.2, Cl: 1.3, K: 0.1, Ca: 30.3, Au: 7.2
	2	C: 39.8, O: 19.9, Na: 1.5, Mg: 0.4, P: 8.3, Cl: 1.7, K: 0.3, Ca: 15.8, Au: 12.3
WPI B5	1	C: 8.8, O: 20.6, Na: 1.1, Mg: 0.6, P: 20.6, Cl: 2.0, K: 0.1, Ca: 39.9, Au: 6.3
	2	C: 48.3, O: 24.0, Na: 1.9, Mg: 0.4, P: 3.8, Cl: 3.0, K: 0.4, Ca: 6.6, Au: 11.6
WPI B15	1	C: 22.1, O: 18.7, Na: 1.2, Mg: 0.2, P: 14.7, Cl: 1.8, K: 0.3, Ca: 34.5, Au: 6.5
	2	C: 17.4, O: 14.5, Na: 1.0, Mg: 0.4, P: 13.5, Cl: 2.0, K: 0.3, Ca: 43.6, Au: 7.3
WPI 0	1	C: 40.8, O: 13.6, Na: 3.3, Mg: 0.3, P: 0.6, Cl: 9.9, K: 0.8, Ca: 1.4, Au: 29.3
	2	C: 40.2, O: 14.9, Na: 3.4, Mg: 0.5, P: 1.2, Cl: 9.7, K: 0.5, Ca: 1.1, Au: 28.5

**Table 8:** Conditions for the determination of the stability of the suspension.

Hydroxyapatite	Weight (g)	V H_2_O (mL)	Measurement Temperature (°C)	Cp (%)
A	0.0440	3	25	1.279
B	0.0404			1.328

**Table 9:** Sedimentation rate for HAp powder in different solutions.

Suspension	HAp	Sedimentation Rate [mm/min]
Distilled water	HAp A	−12.320 ± 1.003
	HAp B	−35.130 ± 2.147
WPI solution	HAp A	−0.050 ± 0.037
	HAp B	−0.110 ± 0.022

## Data Availability

The data that support the findings of this study are contained within the article.
